# Turmeric-Loaded Type A Gelatin Hydrogels: In Vitro Assessment for Oral Wound Management Applications

**DOI:** 10.3390/gels12100927

**Published:** 2026-10-15

**Authors:** Yareth Idalia Ayerim Gutierrez-Jimenez, Rene Garcia-Contreras, Paloma Netzayeli Serrano-Diaz, Juan Carlos Flores-Arriaga, Ravichandran Manisekaran, Carlos A. Jurado, Silvia Rojas-Rueda, Abdullah Almansour, Abdulrahman Alshabib

**Affiliations:** 1 Interdisciplinary Research Laboratory (LII), Nanostructures and Biomaterials Area, National School of Higher Studies (ENES), National Autonomous University of Mexico (UNAM), Leon 37684, Guanajuato, Mexico; 2 Faculty of Stomatology, Autonomous University of San Luis Potosí, San Luis Potosi 78290, San Luis Potosí, Mexico; 3 School of Dental Medicine, Ponce Health Sciences University, Ponce 00716, Puerto Rico; 4 College of Dentistry, Prince Sattam Bin Abdulaziz University, Al-Kharj 11942, Saudi Arabia; 5 Department of Restorative Dental Science, College of Dentistry, King Saud University, Riyadh 11545, Saudi Arabia

**Keywords:** gelatin hydrogel, *Curcuma longa*, oral wound management, human gingival fibroblasts, prostaglandin E_2_

## Abstract

Oral surgical wounds may benefit from local biomaterials capable of delivering bioactive compounds while remaining compatible with gingival cells. This study evaluated type A gelatin hydrogels containing turmeric powder (1.5, 3.1, or 12.5 mg per preparation) and a separate series prepared with a 25 mg/mL turmeric stock in dimethyl sulfoxide (DMSO). The hydrogels were assessed for macroscopic integrity, supernatant absorbance, MTT responses in human gingival fibroblasts (HGFs), prostaglandin E_2_ (PGE_2_), and exploratory microbial response against *Staphylococcus aureus* and *Candida albicans*. Powder-loaded matrices lost macroscopic integrity within approximately 45 min in PBS and 30 min in trypsin. Powder-loaded formulations generally produced MTT signals near control values, whereas several DMSO-stock formulations showed lower signals at later exposures. PGE_2_ did not show consistent dose-dependent suppression after normalization of the corresponding MTT-derived relative metabolic signal. Agar diffusion showed an approximate 2.4 mm clearance beyond the specimen edge against *S. aureus* for the 1.5 mg formulation and none against *C. albicans*; the gelatin control also showed visible clearance, and higher turmeric loadings did not produce measurable zones. Colony recovery decreased across the DMSO-stock formulations, but most plate counts were below the conventional countable range and were therefore interpreted descriptively. Overall, the findings provide an early in vitro characterization of rapidly disintegrating gelatin–turmeric hydrogels and identify matrix stability and assay limitations as major constraints that require further validation.

## 1. Introduction

Oral surgical wounds are exposed to saliva, repeated mechanical loading, food debris, and dense polymicrobial ecosystems. These conditions can shorten the biomaterial residence time and sustain postoperative inflammation or microbial colonization. Local delivery systems that are biocompatible with gingival cells and enable localized delivery of bioactive compounds are attractive adjuncts to conventional postoperative care. Hydrogels are particularly important because their water-rich three-dimensional networks can conform to irregular wound surfaces and serve as carriers of bioactive compounds [1,2].

Gelatin is a collagen-derived biopolymer widely used in drug delivery and in regenerative-medicine research because of its biodegradability, biocompatibility, and processing versatility. Type A gelatin is produced by acid processing and contains cell-interactive peptide sequences that may support cellular adhesion and matrix interactions. Aqueous gelatin solutions form thermoreversible physical gels whose stability is strongly influenced by temperature, concentration, and crosslinking. Non-crosslinked gelatin matrices can soften or dissolve rapidly under physiological conditions; consequently, matrix stability, crosslinking density, and tissue adhesion require characterization before an oral application can be assessed [3,4].

*Curcuma longa* (turmeric) is a multicomponent phytochemical material in which curcumin is a major curcuminoid. It also contains demethoxycurcumin, bisdemethoxycurcumin, turmerones, and other minor constituents that may contribute to its biological activity [5,6]. Curcumin and related turmeric constituents have been associated with modulation of cyclooxygenase-related and other inflammatory pathways in previous studies. However, low aqueous solubility, chemical instability, and limited bioavailability can restrict therapeutic delivery of curcumin and have motivated the development of local and carrier-based delivery strategies [7,8].

Gelatin-based systems containing curcumin have been investigated for oral soft-tissue applications. Gelatin-encapsulated curcumin nanoparticles have previously been evaluated in primary human gingival fibroblasts (HGFs) [9]. Turmeric mouthwash and a 2% whole-turmeric gel have also been investigated for periodontal care [10,11]. These studies provide an oral research rationale, but their formulations and clinical settings differ from the physically gelled matrices examined here. The present preparation contains commercial turmeric powder rather than chemically standardized curcumin; therefore, the resulting formulation should be described as a turmeric-loaded gelatin hydrogel, and its biological effects should not be attributed exclusively to curcumin.

Previous curcumin-containing wound materials include chemically crosslinked polysaccharide systems, micelle-containing hydrogels, and nanocomposite dressings [12,13,14,15], as well as a carboxymethyl cellulose–gelatin formulation investigated for oral tissue repair [16]. These materials differ in polymer composition, crosslinking, and curcumin presentation. The present study does not establish superior performance over these systems. Its narrower contribution is a description of the macroscopic instability of a simple physically gelled type A gelatin formulation and exploratory assay responses that identify constraints on its interpretation.

The objective was to describe macroscopic integrity and supernatant absorbance of powder-loaded type A gelatin matrices, together with exploratory HGF MTT signals, PGE_2_ measurements, and microbial observations. A separate DMSO-stock series was assessed only as complete turmeric–DMSO–gelatin formulations. Because nominal turmeric input and solvent content differed, this was not a controlled comparison of incorporation strategies. The experiments were not designed to demonstrate therapeutic efficacy, controlled release, or wound healing.

## 2. Results and Discussion

### 2.1. Formulation Strategy, Supernatant Leaching, and Macroscopic Integrity

Two formulation series were prepared (Table 1). In the powder series, turmeric was directly incorporated into the gelatin solution at nominal inputs of 1.5, 3.1, or 12.5 mg per preparation. In the stock series, turmeric was dispersed in DMSO at 25 mg/mL and incorporated at 1, 2, 3, 4, or 5 mL, corresponding to nominal turmeric inputs of 25, 50, 75, 100, and 125 mg, respectively. These stock additions corresponded to nominal formulation DMSO fractions of approximately 10, 20, 30, 40, and 50% (*v*/*v*), respectively. Because turmeric input and solvent content varied simultaneously, the powder and stock series were considered separate experimental formulations and were not used for direct comparisons of incorporation strategies, potency, or bioavailability.

**Figure 1:**
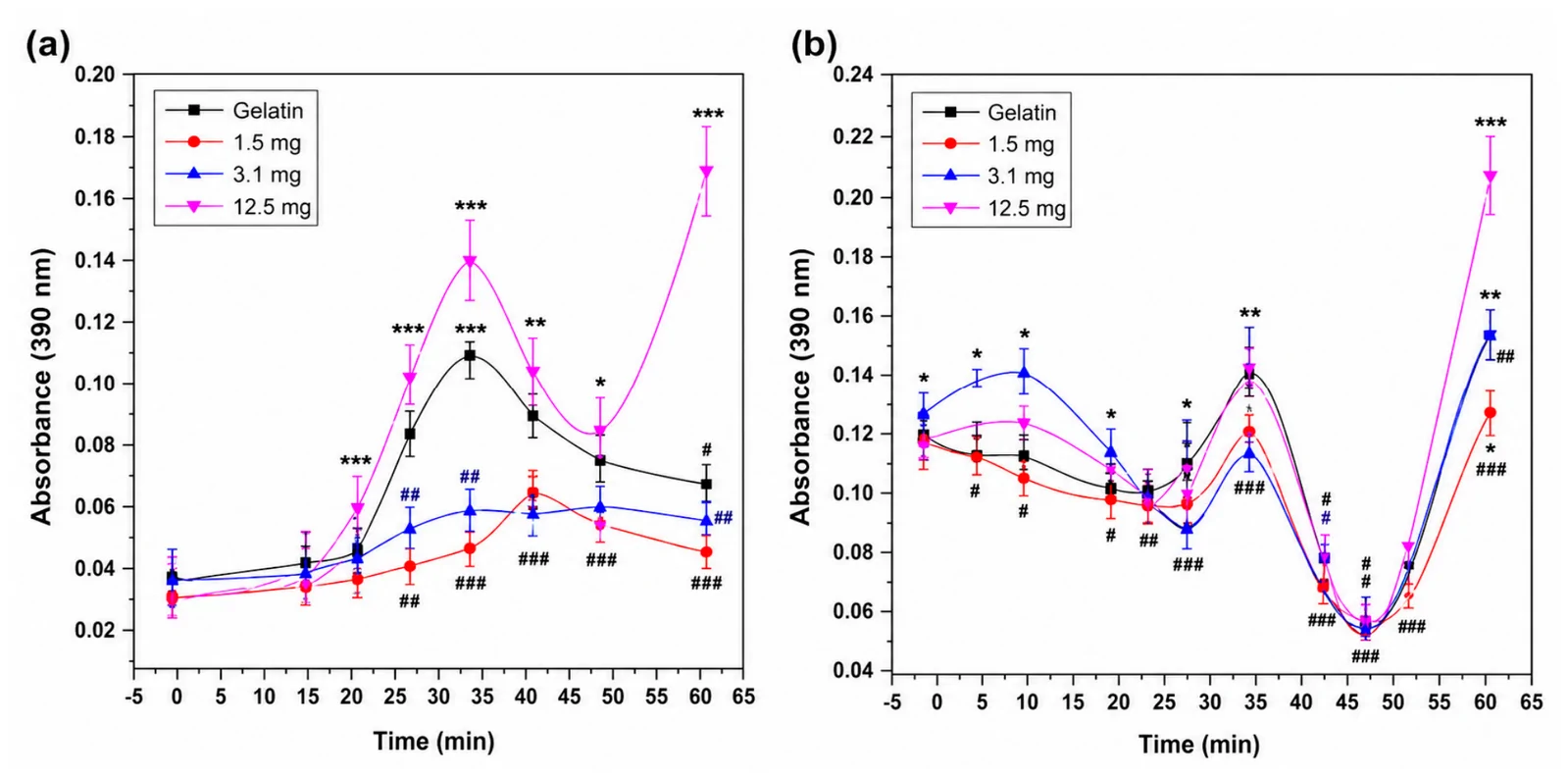
Supernatant absorbance and macroscopic integrity of powder-loaded gelatin–turmeric matrices in PBS and trypsin. (**a**) Exposure in 1 mL of phosphate-buffered saline (PBS, pH 7.4) and (**b**) exposure in 1 mL of 0.005% trypsin. Gelatin-only matrices and matrices loaded with 1.5, 3.1, or 12.5 mg of turmeric were incubated at 37 °C under continuous agitation at 80 rpm. At the indicated time points, the absorbance of the supernatant was measured using UV–Vis spectrophotometry at 390 nm. This signal is considered an indirect measure of material leaching/dispersion rather than gravimetric degradation. Visual observation indicated extensive macroscopic loss of integrity by approximately 45 min in PBS and 30 min in trypsin. Data are presented as the mean ± standard deviation across three independent experimental runs; technical triplicate measurements were averaged within each independent run. Normality was assessed using the Shapiro–Wilk test, followed by two-way analysis of variance (ANOVA) and Tukey’s post hoc multiple-comparison test. Statistical significance was established at *p* < 0.05 versus the gelatin control at the same time point; * *p* < 0.05, ** *p* < 0.01, *** *p* < 0.001, # *p* < 0.05, ## *p* < 0.01, and ### *p* < 0.001 versus the 12.5 mg group at the same time point.

The powder-loaded matrices lost macroscopic integrity within approximately 45 min in PBS and 30 min in trypsin (Figure 1). The supernatant absorbance at 390 nm increased in several groups. This nonspecific signal may include contributions from turmeric-derived chromophores, suspended particles, and other matrix-associated material. It does not quantify remaining dry mass, polymer degradation, or cumulative phytochemical release.

For this preparation, loss of macroscopic integrity is a formulation constraint rather than evidence of a useful clinical residence time. More structured curcumin-containing matrices have been investigated using other polymers, crosslinking approaches, and nanocomposite designs [12,13,14,15,17,18]. Their reported properties cannot be transferred to the present physical gel. Improving stability would require new formulation experiments and independent evaluation of material and biological properties.

### 2.2. MTT Assay Signals in Human Gingival Fibroblast Cultures

**Figure 2:**
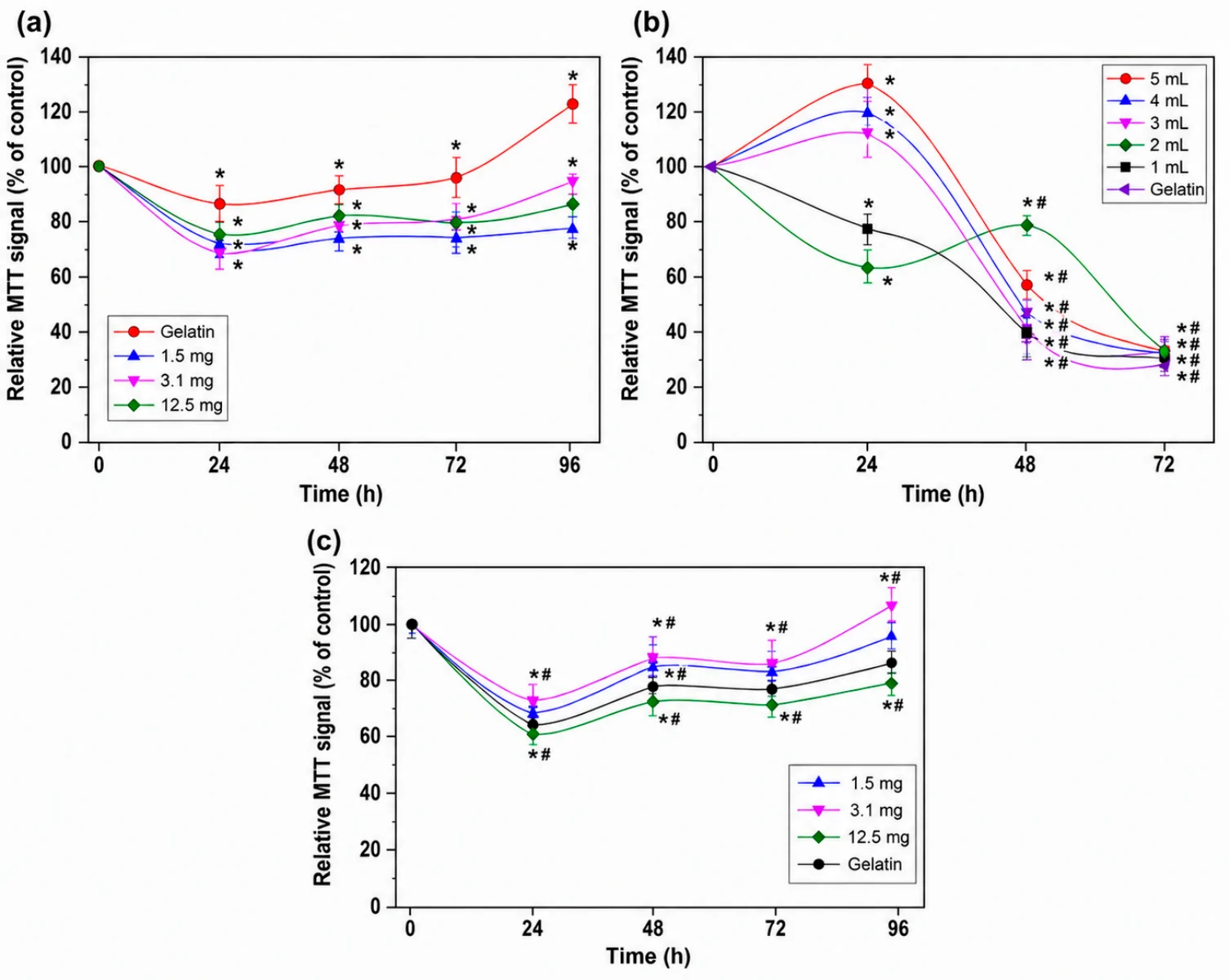
Metabolic activity of human gingival fibroblasts exposed to gelatin–turmeric hydrogels. Measurements were obtained at 24, 48, 72, and 96 h of contact. (**a**) Exposure initiated with powder-loaded matrices containing nominal inputs of 1.5, 3.1, or 12.5 mg per preparation. (**b**) Exposure initiated with matrices prepared using 1–5 mL of 25 mg/mL turmeric stock in DMSO. (**c**) Indirect exposure to extracts prepared by incubating the powder-loaded matrices in MEM culture medium for 24 h at 37 °C under continuous agitation at 80 rpm. Gelatin-only matrices served as material controls. HGFs were seeded at approximately 2 × 10^5^ cells/mL. Formazan absorbance was measured at 570 nm and expressed as relative MTT signal (% of control), not as an independently validated direct measure of cell viability or cytocompatibility. No formulation-matched acellular turmeric blanks or stock-series DMSO-matched controls were included. Data are presented as the mean ± standard deviation across three independent experimental runs; technical triplicate measurements were averaged within each independent run. Normality test was assessed by the Shapiro–Wilk test, followed by two-way analysis of variance and Tukey’s post hoc multiple-comparison test. Statistical significance was established at *p* < 0.05. * *p* < 0.05 versus the gelatin control at the same exposure time; # *p* < 0.05 versus the corresponding 24 h value within the same formulation.

Direct and indirect MTT measurements were obtained at 24, 48, 72, and 96 h (Figure 2). Powder-loaded formulations generally showed MTT signals close to the corresponding gelatin control over the evaluation period. These measurements reflect cellular metabolic reduction under the tested exposure conditions rather than direct evidence of increased proliferation, regeneration, or tissue-repair activity. The cytotoxicity was interpreted according to ISO 10993-5 [19].

The DMSO-stock series showed a distinct time-dependent pattern, with several formulations exhibiting lower MTT signals at later exposure periods. The five stock formulations contained nominal DMSO fractions of approximately 10–50% (*v*/*v*) before exposure. Because matched gelatin + DMSO controls were not available for each solvent fraction, these responses are interpreted at the level of the complete turmeric–DMSO–gelatin formulation and cannot be assigned independently to turmeric or DMSO.

Direct contact describes the initial exposure condition rather than persistence of an intact hydrogel throughout the 24–96 h observation period. Because rapid disintegration was demonstrated in PBS and trypsin, later measurements may represent cellular exposure to dissolved or dispersed matrix components rather than sustained contact with an intact gel.

Direct contact describes how exposure was initiated, not the persistence of a hydrogel throughout 24–96 h. Consequently, later measurements must be interpreted as exposure to evolving matrix contents, potentially including dissolved gelatin and dispersed turmeric. These measurements cannot demonstrate recovery of cells on an intact matrix. A material contribution from serum-dependent curcuminoid solubility is also plausible on the basis of earlier work [20], but was not measured here.

### 2.3. PGE_2_ Response Under Basal and IL-1β-Stimulated Conditions

**Figure 3:**
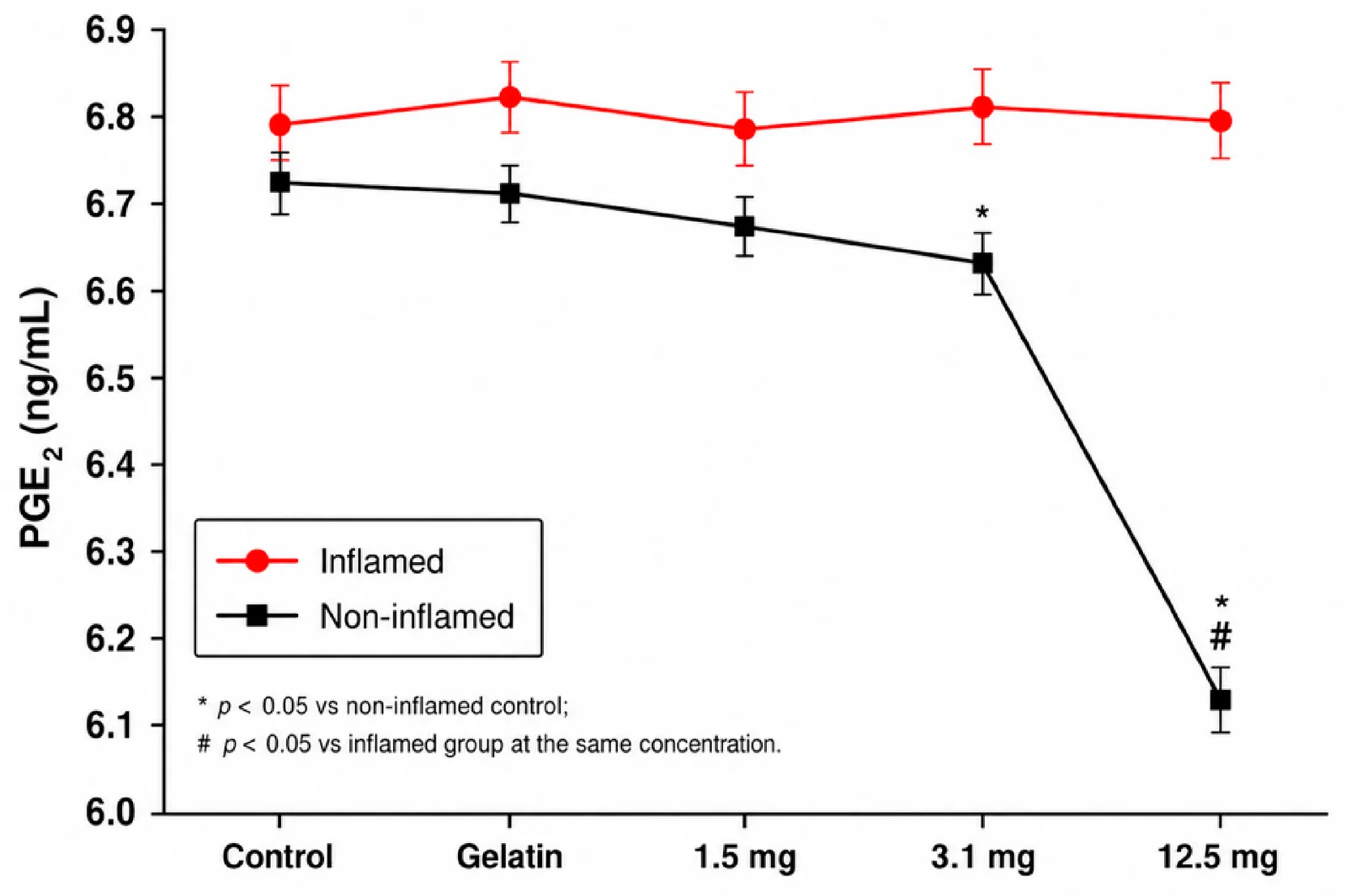
PGE_2_ concentrations in human gingival fibroblasts under basal and IL-1β-stimulated conditions after exposure to turmeric-loaded gelatin matrices. HGFs were cultured in direct contact with gelatin-only matrices ‘Control’ denotes HGFs cultured without matrix exposure, whereas ‘Gelatin’ denotes contact with the gelatin-only matrix; the remaining groups contained 1.5, 3.1, or 12.5 mg of turmeric powder. Cells were pretreated with interleukin-1β (IL-1β; 3 ng/mL) for 3 h before matrix exposure; parallel cultures without IL-1β were evaluated as non-inflamed groups. After 24 h, culture supernatants were collected and PGE_2_ was measured using a commercial ELISA kit at 405 nm. Concentrations were calculated from the standard curve and normalized to the corresponding MTT-derived relative metabolic signal. Data are presented as the mean ± standard deviation across three independent experimental runs; technical triplicate measurements were averaged within each independent run. Normality was assessed by the Shapiro–Wilk test, followed by two-way analysis of variance and Tukey’s post hoc multiple-comparison test. Statistical significance was established at *p* < 0.05. * *p* < 0.05 versus the non-inflamed control; # *p* < 0.05 versus the corresponding inflamed group at the same turmeric concentration.

PGE_2_ was evaluated in supernatants after 24 h of matrix exposure, with or without preceding IL-1β stimulation at 3 ng/mL for 3 h (Figure 3). PGE_2_ concentrations were calculated from the ELISA standard curve and normalized to MTT-derived relative metabolic signal before group comparisons. Following normalization, the data did not demonstrate a consistent dose-dependent suppression of PGE_2_ under IL-1β-stimulated conditions. Individual between-group differences were observed, but the overall pattern did not support a progressive inhibitory response with increasing nominal turmeric content.

Pharmacological suppression control was not included; therefore, these findings are interpreted as the PGE_2_ response observed under the tested experimental conditions rather than as validation of anti-inflammatory efficacy, and the lower signal observed in the basal 12.5 mg group should not be considered evidence of per-cell suppression [5,6,21].

### 2.4. Exploratory Microbial Observations

**Figure 4:**
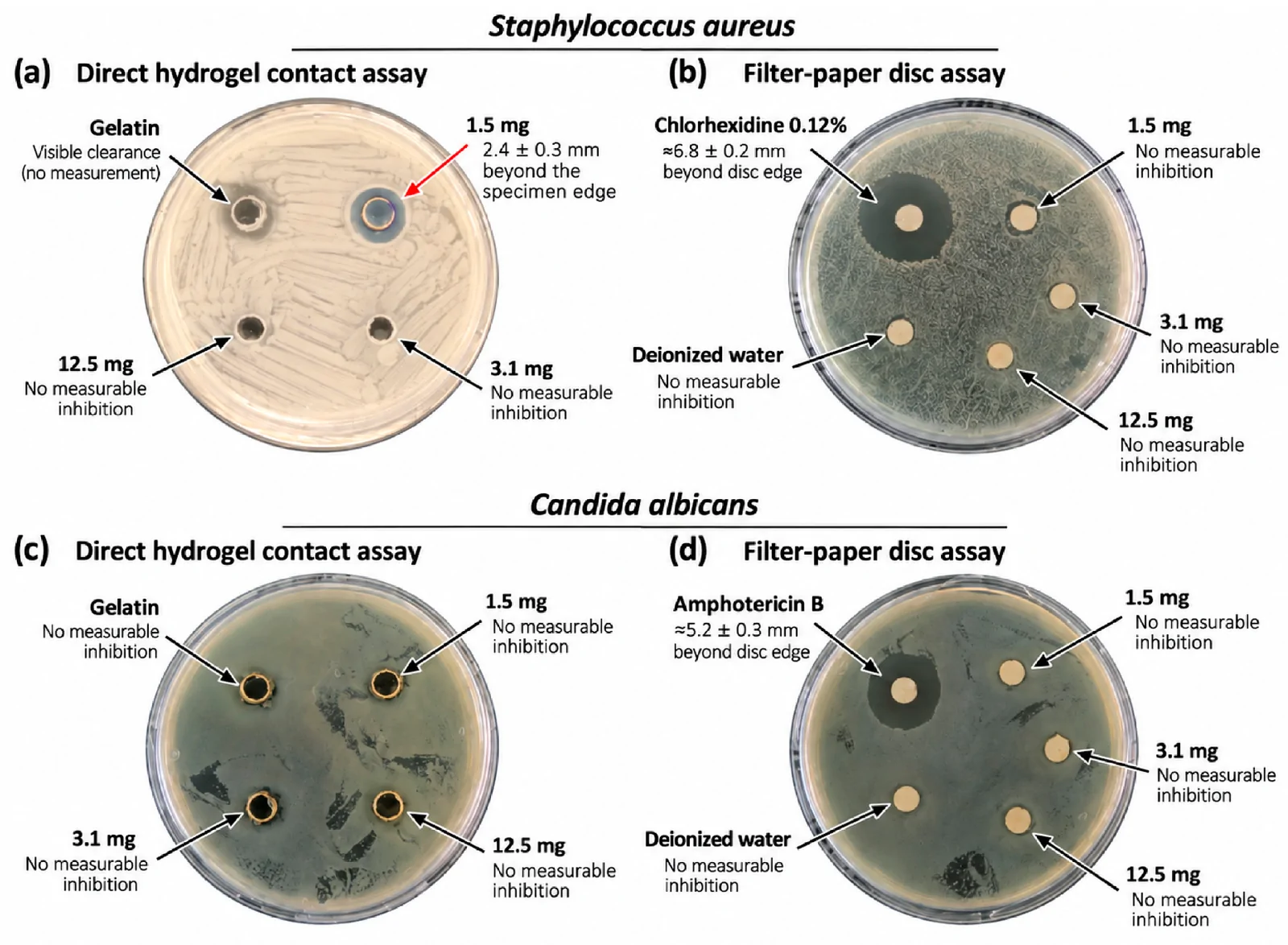
Agar diffusion observations for turmeric-loaded gelatin matrices. Representative plates show the response of *Staphylococcus aureus* in the direct hydrogel-contact assay (**a**) and filter-paper disc assay (**b**), and of *Candida albicans* in the direct hydrogel-contact assay (**c**) and filter-paper disc assay (**d**), after 24 h of incubation. Gelatin-only matrices and matrices loaded with 1.5, 3.1, or 12.5 mg of turmeric powder were evaluated. Microbial suspensions were adjusted to a 0.5 McFarland standard and inoculated onto Mueller–Hinton agar for *S. aureus* or Sabouraud dextrose agar for *C. albicans*. Chlorhexidine 0.12% and amphotericin B were used as positive controls for *S. aureus* and *C. albicans*, respectively and deionized water was used as a negative control. A 2.4 ± 0.3 mm inhibition-zone measurement was recorded for *S. aureus* in association with the 1.5 mg formulation, whereas a visible but unquantified clearance was also observed around the gelatin-only control and no measurable inhibition zone was detected for the 3.1 or 12.5 mg formulations. The chlorhexidine positive control produced an inhibition zone of 6.8 ± 0.2 mm beyond the disc edge. Against *C. albicans*, no measurable inhibition was observed for the gelatin-only control or any turmeric-loaded formulation, whereas amphotericin B produced an inhibition zone of 5.2 ± 0.3 mm beyond the disc edge. Higher powder loadings did not produce a corresponding dose-dependent response; therefore, this isolated observation is interpreted cautiously and cannot be attributed specifically to turmeric or considered evidence of turmeric-specific antibacterial potency.

Agar diffusion testing showed a measurable inhibition zone against *S. aureus* for the 1.5 mg turmeric-loaded formulation (2.4 ± 0.3 mm beyond the specimen edge). A visible but unquantified clearance was also observed around the gelatin control, whereas no measurable inhibition was detected for the 3.1 or 12.5 mg formulations. The chlorhexidine 0.12% positive control produced the largest inhibition zone of 6.8 ± 0.2 mm and the amphotericin B inhibition zone of 5.2 ± 0.3 mm beyond the disc edge, while deionized water showed no measurable inhibition, respectively. Against *C. albicans*, no measurable inhibition was observed for gelatin or any turmeric-loaded formulation, whereas amphotericin B produced an estimated inhibition zone of approximately 5.2 ± 0.3 mm beyond the disc edge (Figure 4). Because the gelatin control also showed visible clearance and higher turmeric loadings did not produce a monotonic response, the isolated 1.5 mg observation cannot be attributed specifically to turmeric.

**Figure 5:**
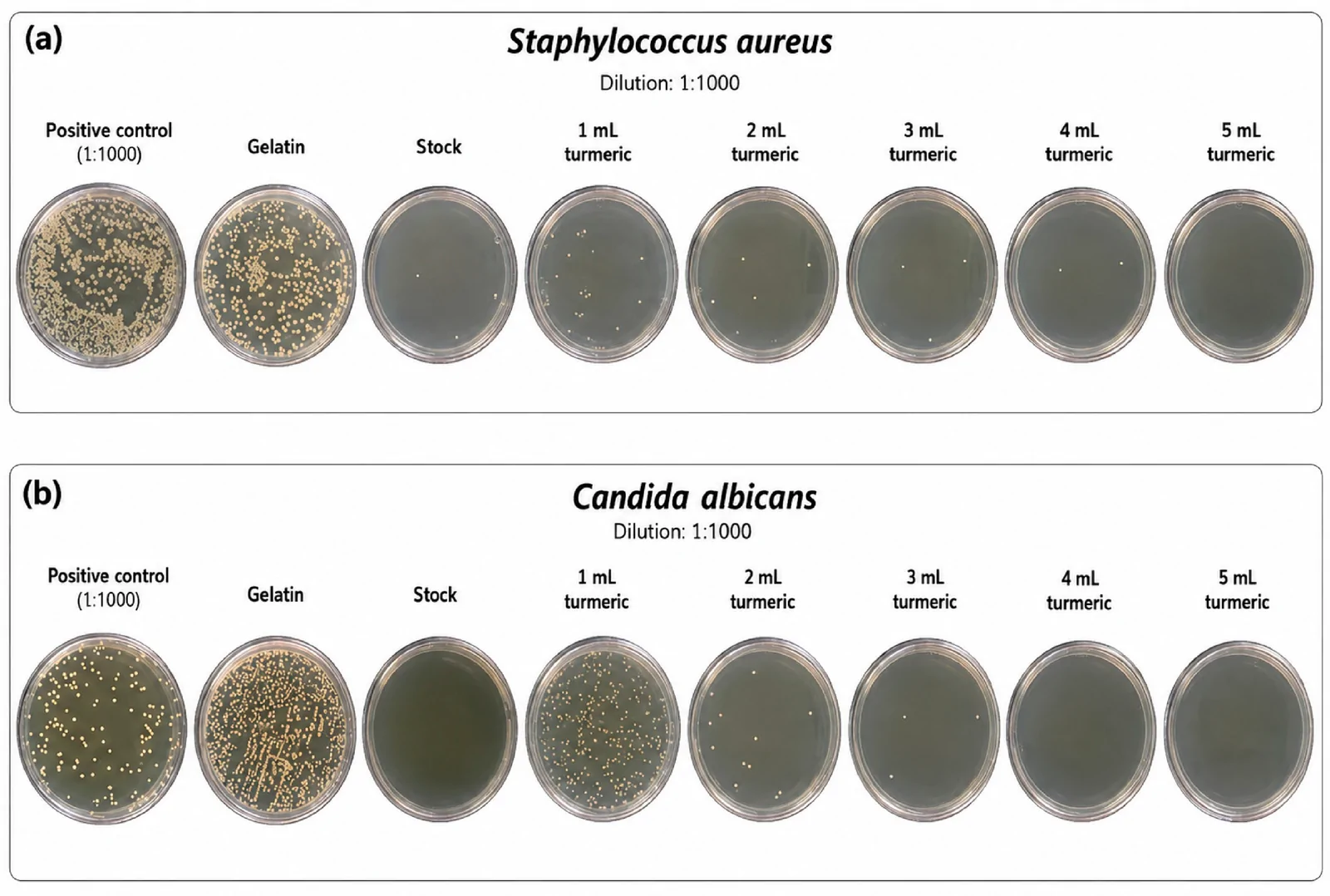
Colony-recovery assessment after microbial exposure to gelatin matrices containing DMSO-based turmeric stock. Representative culture plates show (**a**) *Staphylococcus aureus* and (**b**) *Candida albicans* recovered after 24 h of exposure to matrices prepared with 1, 2, 3, 4, or 5 mL of a 25 mg/mL turmeric stock in DMSO. Following exposure, microbial suspensions were serially diluted, and 15 µL of the 1:1000 dilution was plated on Mueller–Hinton agar for *S. aureus* or Sabouraud dextrose agar for *C. albicans*. The approximate recovered concentrations ranged from 0.93 ± 0.07 to 0.22 ± 0.04 × 10^6^ CFU/mL for *S. aureus* and from 10.35 ± 0.23 to 0.25 ± 0.14 × 10^6^ CFU/mL for *C. albicans* across the 1–5 mL formulation series. Overall differences among formulations were detected for *S. aureus* (Kruskal–Wallis, df = 4, *p* = 0.038) and *C. albicans* (Kruskal–Wallis, df = 4, *p* = 0.039); however, most corresponding colony counts were below the conventional approximately 30–300 colony countable range. Therefore, these statistical results and recovered concentrations should be interpreted as exploratory and with caution. These omnibus tests do not identify specific pairwise differences or establish a statistically supported dose–response relationship. Because the nominal DMSO content increased concurrently across formulations, the observed changes in microbial recovery reflect the response to the complete turmeric–DMSO–gelatin system and cannot be attributed specifically to turmeric. No vehicle-referenced log reduction, MIC, or MBC endpoint was determined.

For the stock-loaded formulation series, mean *S. aureus* colony counts progressively decreased from 14.0 ± 1.0 colonies in the 1 mL group to 3.3 ± 0.6 colonies in the 5 mL group, whereas *C. albicans* counts decreased from 155.3 ± 3.5 to 3.7 ± 2.1 colonies across the same formulation range (Figure 5). Based on the reported 1:1000 dilution and 15 µL plated volume, the corresponding approximate recovered concentrations were (0.93 ± 0.07), (0.53 ± 0.13), (0.33 ± 0.23), (0.29 ± 0.10), and (0.22 ± 0.04) × 10^6^ CFU/mL for *S. aureus*, and (10.35 ± 0.23), (1.22 ± 0.34), (0.80 ± 0.58), (0.31 ± 0.04), and (0.25 ± 0.14) × 10^6^ CFU/mL for *C. albicans*, for the 1–5 mL groups, respectively. Overall differences among formulations were detected for both microorganisms (*S. aureus*: Kruskal–Wallis, df = 4, *p* = 0.038; *C. albicans*: Kruskal–Wallis, df = 4, *p* = 0.039), indicating formulation-dependent variation in microbial recovery. However, except for the *C. albicans* 1 mL group, the mean colony counts were below the conventional approximately 30–300 colony countable range. Therefore, the Kruskal–Wallis results and calculated CFU/mL values should be considered exploratory and interpreted cautiously rather than as robust quantitative evidence of differences among formula. These omnibus tests do not identify specific pairwise differences or demonstrate a statistically supported monotonic dose–response relationship. Descriptively, colony recovery from the 1 mL to the 5 mL formulation decreased by 76.4% for *S. aureus* and 97.6% for *C. albicans*. In addition, because the nominal DMSO content increased concurrently across the stock formulations, the observed reduction in microbial recovery cannot be attributed specifically to turmeric. Therefore, these findings should be interpreted as formulation-associated differences in microbial recovery rather than evidence of turmeric-specific efficacy, enhanced bioavailability, or determination of MIC or MBC endpoints [22].

### 2.5. Study Relevance

Our initial studies of a physically gelled gelatin–turmeric matrix were aimed at evaluating its preliminary in vitro behavior, rather than establishing its suitability as an oral wound dressing or as a regenerative scaffold. The gelatin matrix provides a hydrated, biodegradable environment for incorporated compounds [1,2,3,4]; however, localized therapeutic effects were not established in this study. The experiments described here are preliminary in nature and were designed to assess a limited number of properties of the matrix, i.e., MTT assay signals in HGF cultures, PGE_2_ responses, the gross appearance of the matrix, supernatants absorbances at 390 nm, and the preliminary microbiological observations.

The turmeric powder used in this study is a multicomponent phytochemical source rather than a chemically standardized curcumin preparation. The major phytochemicals found in the plant *Curcuma longa* are the curcuminoids (curcumin, demethoxycurcumin, and bisdemethoxycurcumin) and the volatile compounds (ar-turmerone, α-turmerone, and β-turmerone) [23,24,25,26,27]. However, the commercial turmeric powder used in these studies was not quantitatively analyzed for its phytochemical composition. Thus, the amount of turmeric powder added to the matrix in mg did not translate into a corresponding amount of curcuminoids. Consequently, the observed assay responses cannot be attributed to curcumin or to any other specific phytochemicals and cannot be assigned to a specific phytochemical constituent.

The powder-loaded matrices quickly lost their macroscopic integrity within approximately 45 min in PBS and 30 min in trypsin. This time frame is a material limitation rather than an advantageous residence time for clinical applications. These observations do not establish intraoral residence time or a handling advantage over existing oral formulations, such as chlorhexidine gels. The present study did not quantify cumulative phytochemical release, dissolution, mass balance, or chemical stability in the exposure media. Therefore, macroscopic disintegration and supernatant absorbance at 390 nm should not be interpreted as evidence of controlled or bioavailable phytochemical release. Future formulations will require evaluation of an appropriate crosslinking strategy to increase matrix stability, together with evaluation of adhesion strategies and independent characterization of physicochemical and biological properties using approaches relevant to more structured gelatin-based systems, including gelatin methacryloyl (GelMA) materials [10,11,12,13,22].

The MTT measurements obtained with the powder-loaded formulations reflect relative cellular metabolic reduction under the tested exposure conditions and do not provide direct evidence of increased proliferation, fibroblast regeneration, cytocompatibility, or tissue-repair activity. Furthermore, the persistence of an intact matrix in the culture medium was not verified; therefore, later exposure periods may involve dissolved or dispersed matrix constituents.

PGE_2_ concentrations were calculated from the ELISA standard curve and normalized to the corresponding MTT-derived relative metabolic signal before group comparisons. Following normalization, no consistent dose-dependent suppression of the IL-1β-stimulated response was observed. Because a pharmacological suppression control was not included, these findings describe the PGE_2_ response under the tested conditions but do not establish anti-inflammatory efficacy. Published studies have associated turmeric-derived compounds with modulation of cyclooxygenase- and NF-κB-related signaling [5,6,16,24,25,26], but these pathways were not directly evaluated in the present study and were not used to infer a specific mechanism of action.

The microbiological observations should likewise be interpreted cautiously. Agar diffusion showed only an isolated clearance around the lowest powder-loading formulation, while the gelatin control also showed visible clearance and higher turmeric loadings did not produce a monotonic response. In the DMSO-stock series, colony-count analysis demonstrated formulation-dependent differences in microbial recovery. Most colony counts were below the conventional countable range, and because DMSO-matched controls were absent, the relative contributions of turmeric and DMSO to the observed microbial responses cannot be distinguished. Moreover, the stock formulations contained substantially greater nominal turmeric inputs than the powder formulations, in addition to increasing DMSO volumes. Consequently, the powder and stock series should be considered separate exploratory formulations and cannot be used to infer improved bioavailability, greater antimicrobial efficacy, or superiority of one incorporation strategy over the other. Although gelatin–curcumin biomaterials have previously demonstrated antibacterial and wound-related biological activity [10,11,12,13,17,20,22,28], such effects require independent quantitative validation in the present system.

Established oral wound management approaches, including chlorhexidine gels, hyaluronic-acid gels [29], and platelet-rich fibrin [30], are cited only to provide clinical context; their retention mechanisms, composition, and clinical evidence differ substantially from the present formulation, and no direct comparison or superiority is implied.

Future studies should therefore chemically characterize the principal potentially active constituents using validated HPLC/LC–MS or GC–MS methods, determine incorporation efficiency, content uniformity, filtration-associated losses, and batch-to-batch variability, and establish quantitative release kinetics under biologically relevant conditions. These chemical measurements should subsequently be correlated with orthogonal cytocompatibility assessments, pharmacologically controlled inflammatory assays, quantitative antimicrobial outcomes, and saliva-conditioned polymicrobial oral biofilm models.

### 2.6. Study Limitations

Formulation and chemical limitations: The turmeric powder was not chemically standardized, and incorporation efficiency, phytochemical release, content uniformity, mass balance, chemical stability, mechanical properties, and mucoadhesion were not quantified. The powder and DMSO-stock series differed in both turmeric input and solvent content and therefore cannot be compared as dose-matched or vehicle-matched systems.

Biological-assay limitations: HGFs were obtained from a single donor, and only three independent runs were available. No orthogonal cytocompatibility assay, pharmacological suppression control, or dedicated formulation-matched acellular blanks were included. Consequently, MTT results should be interpreted as relative metabolic signals and PGE_2_ findings as exploratory responses rather than as evidence of validated cytocompatibility or anti-inflammatory efficacy.

Microbiological and translational limitations: Antimicrobial testing was restricted to single planktonic strains, agar diffusion measurements were derived from archived images, and most colony counts fell below the conventional countable range. DMSO-matched vehicle controls were not included, and testing was performed without control-referenced log reductions, MIC/MBC determination, saliva conditioning, or polymicrobial biofilm models. These constraints substantially limit quantitative and clinical interpretation.

Overall, these findings should be considered preliminary proof-of-concept evidence requiring further physicochemical and biological validation.

## 3. Conclusions

Type A gelatin hydrogels containing turmeric provided preliminary in vitro information on relative MTT assay signals in HGF cultures, PGE_2_ responses, matrix behavior, and formulation-associated microbiological observations. The PGE_2_ data do not demonstrate a definitive anti-inflammatory effect, and the microbiological observations obtained with DMSO-stock formulations cannot be separated from possible vehicle effects. Powder-loaded matrices showed rapid macroscopic loss of integrity under the tested conditions, while quantitative phytochemical content, incorporation efficiency, release kinetics, mechanical behavior, mucoadhesion, and antimicrobial efficacy were not established. Further development requires standardized phytochemical characterization, DMSO vehicle-matched controls, orthogonal cytocompatibility assays, quantitative release and antimicrobial testing, improved material stability and adhesion, and validated saliva-conditioned polymicrobial oral wound/biofilm models.

## 4. Materials and Methods

### 4.1. Study Design

In this descriptive in vitro study, three independent runs with technical triplicate measurements averaged within each run were performed for two experimentally distinct series of physically gelled type A gelatin–turmeric matrices: turmeric powder was directly incorporated into the gelatin matrix, and a turmeric stock dispersed in DMSO was used. The series were neither dose-matched nor vehicle-matched and were not used to compare incorporation strategies. The run, rather than each technical measurement, was the unit used for statistical analysis. For HGFs, independent runs used cultures derived from the same donor.

### 4.2. Preparation of Powder-Loaded Type A Gelatin Hydrogels

Sterile deionized water was heated with agitation. Type A gelatin (Sigma-Aldrich, St. Louis, MO, USA) and commercial turmeric powder (Golden Plant’s, Nezahualcoyotl State of Mexico, Mexico) were placed in labeled vessels corresponding to 0, 1.5, 3.1, and 12.5 mg turmeric. For each group, 2 g of gelatin was mixed with 10 mL of heated water (20% *w*/*v*) and agitated until homogeneous. Six milliliters of each preparation were transferred to Petri dishes, cooled at room temperature to form physical gels, punched into specimens, and stored under refrigeration. Quantitative specimen thickness/mass and gravimetric loading were not recorded for the powder series; therefore, the reported turmeric values represent the nominal input.

### 4.3. Preparation of Turmeric-Stock-Loaded Hydrogels

A turmeric stock was prepared in DMSO at 25 mg/mL (Karal, Leon, Guanajuato, Mexico) and filtered before incorporation. Type A gelatin (2 g per formulation) was dissolved in heated sterile deionized water. Stock volumes of 1, 2, 3, 4, or 5 mL were added, corresponding to nominal turmeric inputs of 25, 50, 75, 100, and 125 mg, respectively. Based on the documented formulation volumes, these stock additions corresponded to nominal DMSO fractions of approximately 10, 20, 30, 40, and 50% (*v*/*v*), respectively. Six milliliters of each formulation were transferred to Petri dishes, cooled to form gels, refrigerated, and punched into 5-mm-diameter specimens. The effective DMSO concentration released during cellular or microbiological exposure was not determined, and turmeric loss during filtration was not quantified. In addition, matched DMSO-only gelatin controls were not included; therefore, potential DMSO-related effects cannot be fully separated from turmeric-specific effects in the stock-series experiments.

### 4.4. Supernatant Leaching and Macroscopic Integrity Assessment

For each specimen-level incubation, a powder-loaded specimen was placed in a microcentrifuge tube containing 1 mL PBS (pH 7.4) or 1 mL 0.005% trypsin (Sigma-Aldrich, St. Louis, MO, USA). The samples were incubated at 37 °C and 80 rpm for 0–60 min. Supernatants were collected and analyzed by UV-visible spectrophotometry at 390 nm (Thermo Fisher Scientific, Vantaa, Finland). The 390 nm measurement was not a validated gravimetric degradation assay, and no remaining dry mass measurements or cumulative phytochemical-release analyses were performed. This experiment was conducted only for the powder-loaded series and was repeated in three independent experimental runs; technical triplicate absorbance measurements within each run were averaged before analysis. Macroscopic loss of matrix integrity was assessed visually.

### 4.5. Primary Human Gingival Fibroblast Culture

Primary HGFs were previously established from a gingival tissue biopsy collected during third-molar surgery from a 21-year-old donor after written informed consent. The original tissue collection protocol was endorsed by the Internal Bioethics Committee of ENES Leon, UNAM (registration CE_16/004_SN), as previously reported [31]. Tissue was sectioned into approximately 1 × 1 mm explants and cultured in minimum essential medium (Sigma-Aldrich, St. Louis, MO, USA) supplemented with 20% fetal bovine serum (Sigma-Aldrich, St. Louis, MO, USA), 1% penicillin–streptomycin (PenStrep, Sigma-Aldrich, St. Louis, MO, USA), and 1% glutamine (GlutaMAX, Sigma-Aldrich, St. Louis, MO, USA). Cultures were maintained at 37 °C, 5% CO_2_, and 95% relative humidity, with medium replacement every three days after the first week. Cells were passaged at approximately 80% confluence using 0.05% trypsin-EDTA. The present experiments used these previously established HGF cultures.

### 4.6. Direct and Indirect MTT Assays

Hydrogel specimens were subjected to UV-C irradiation at 253.7 nm (52 μW/cm^2^) at 5 cm for 20 min before cell exposure. Because sterility and phytochemical stability after UV-C treatment were not independently validated, this step is described as a UV-C pretreatment rather than validated sterilization. For exposure initiated by direct contact, HGFs were seeded at a reported density of 2 × 10^5^ cells/mL onto the matrix formulations. For indirect exposure, powder-loaded matrices were agitated in complete culture medium for 24 h at 37 °C and 80 rpm, and the resulting extracts were added to HGFs in 96-well plates. Direct and indirect cultures were incubated for 24, 48, 72, and 96 h. MTT (Sigma-Aldrich, St. Louis, MO, USA) was added at 0.2 mg/mL; formazan was dissolved in DMSO and absorbance was assessed at 570 nm using a Multiskan Go microplate reader (Thermo Fisher Scientific, Vantaa, Finland). Relative MTT assay signals were expressed against the corresponding cellular control. Acellular turmeric-containing blanks were used to correct absorbance before relative MTT signals were calculated, reducing formulation-associated optical interference.

### 4.7. PGE_2_ ELISA

HGFs were cultured in direct contact with the powder-loaded hydrogels for 24 h. A pro-inflammatory condition was induced using IL-1β at 3 ng/mL (R&D Systems, Minneapolis, MN, USA) for 3 h before hydrogel exposure. Parallel cultures without IL-1β were evaluated under non-inflamed conditions. Culture supernatants were collected and PGE_2_ was quantified using a commercial ELISA kit (Enzo Life Sciences, New York, NY, USA) according to the manufacturer’s instructions, with absorbance measured at 405 nm. PGE_2_ concentrations were calculated from the ELISA standard curve and subsequently normalized to the corresponding MTT-derived relative metabolic signal.

### 4.8. Agar Diffusion Assay

Young cultures of *Staphylococcus aureus ATCC 25923* and *Candida albicans ATCC 10231* (American Type Culture Collection, ATCC, Manassas, VA, USA) were prepared on Mueller-Hinton agar (BD Bioxon, Cuautitlan Izcalli, State of Mexico, Mexico) and Sabouraud dextrose agar (BD Bioxon, Estado de México, Mexico), respectively. Three to five colonies of each microorganism were suspended in 10 mL of 0.8% sodium chloride solutions, vortexed for 10 s, and adjusted to a turbidity equivalent to 0.5 McFarland using a densitometer.

A 200 μL aliquot of each standardized suspension was spread on the corresponding agar medium. Powder-loaded hydrogel specimens (0, 1.5, 3.1, and 12.5 mg) were placed on the inoculated plates. Chlorhexidine 0.12% (Dentscare Ltda., FGM, Joinville, SC, Brazil), and amphotericin B (Sigma-Aldrich, St. Louis, MO, USA) served as positive controls for *S. aureus* and *C. albicans*, respectively; deionized water corresponded to a negative control. Plates were incubated for 24 h at 37 °C, and inhibition zones were measured.

### 4.9. Hydrogel-Contact Growth Assessment and Colony Recovery

Overnight cultures of *S. aureus* in Mueller-Hinton broth and *C. albicans* in Sabouraud dextrose broth were adjusted to 0.5 McFarland and diluted into working suspensions. Stock-loaded matrix specimens obtained from preparations containing to 1–5 mL DMSO-based turmeric stock were placed in 96-well plates, and 100 μL of microbial suspension was added to each well. The plates were incubated at 37 °C for 24 h. Following exposure, serial dilutions were prepared in the corresponding broth, and 15 μL aliquots of the 1:1000 dilution were plated on Mueller–Hinton agar for *S. aureus* or Sabouraud dextrose agar for *C. albicans* and incubated for 24 h. Colonies were counted after incubation. Viable counts were expressed as CFU/mL according to the equation CFU/mL = *n* × dilution factor/plated volume (mL), corresponding in the present assay to *n* × 10^3^/0.015, where *n* represents the number of recovered colonies. Because the turmeric stock was prepared in DMSO, the solvent contribution was considered as part of the formulation background when interpreting differences in colony recovery.

### 4.10. Statistical Analysis

Normality was assessed using the Shapiro–Wilk test. Homogeneity of variance was assessed for parametric analyses before ANOVA. The experimental design comprised three independent runs with technical triplicate measurements within each run. Technical triplicates were averaged within each independent run and were not treated as independent experimental observations. Formulation and time were evaluated using two-way ANOVA for time-course experiments, whereas inflammatory condition and formulation were evaluated using two-way ANOVA for PGE_2_ data, followed by Tukey’s post hoc multiple-comparison test. Tukey adjustment was applied within each corresponding ANOVA comparison family to control for multiplicity. Principal ANOVA effects and interactions were reported with their corresponding test statistics and exact *p* values when relevant. Because colony-count data were discrete and the number of independent experiments was small (*n* = 3 per formulation), differences among turmeric-stock groups were analyzed separately for *S. aureus* and *C. albicans* using the Kruskal–Wallis test. For these analyses, degrees of freedom and exact *p* values are reported. No pairwise post hoc comparisons or formal trend analyses were performed for colony-count data; therefore, Kruskal–Wallis results were interpreted as overall differences among formulations and not as evidence of specific pairwise differences or a monotonic dose–response relationship. Statistical analyses were performed using SPSS (Version 21, IBM Corp., Armonk, NY, USA). Statistical significance was set at *p* < 0.05.

## Figures and Tables

**Table 1:** Experimental gelatin–turmeric formulation groups.

Series	Group	Turmeric Presentation	Nominal Turmeric Input	Purpose
Powder	GEL	None	0 mg	Gelatin control
Powder	T1.5	Powder	1.5 mg per preparation	Low loading
Powder	T3.1	Powder	3.1 mg per preparation	Intermediate loading
Powder	T12.5	Powder	12.5 mg per preparation	High loading
Stock	S1–S5	25 mg/mL turmeric stock in DMSO	S1 = 25 mg; S2 = 50 mg; S3 = 75 mg; S4 = 100 mg; S5 = 125 mg (nominal turmeric input)	Exploratory DMSO-stock series; not dose-matched to powder series
Stock	GEL-stock	None	0 mL stock	Gelatin control without turmeric; not a DMSO vehicle-matched control

## Data Availability

The data supporting the findings of this study are available from the corresponding author upon reasonable requests.
